# Personality, Humor Styles and Happiness: Happy People Have Positive Humor Styles

**DOI:** 10.5964/ejop.v12i3.1160

**Published:** 2016-08-19

**Authors:** Thomas E. Ford, Shaun K. Lappi, Christopher J. Holden

**Affiliations:** a Western Carolina University, Cullowhee, NC, USA; b Oakland University, Rochester, MI, USA; Department of Psychology, University of Western Ontario, London, Canada

**Keywords:** happiness, subjective well-being, personality, humor styles

## Abstract

The present study examined the relationships between four personality traits, humor styles, and happiness. Replicating previous research, happiness was positively correlated with four personality traits: extraversion, locus of control, self-esteem, and optimism. Further, happiness positively related to self-enhancing and affiliative humor styles; it related negatively to self-defeating and aggressive humor styles. Thus, happy people habitually engage in positive uses of humor and avoid engaging in negative uses of humor in daily life. We also found support for our hypothesis. People high in extraversion, locus of control, self-esteem, and optimism are happier because they engage in positive humor in daily life.

Jordyn Wieber had this to say after winning a gold medal in the women’s gymnastics team competition in the 2012 Summer Olympics: “To have this gold medal around my neck right now is an indescribable feeling. I'm the happiest person right now.” It’s easy to understand why Jordyn Wieber would be happy after such a monumental achievement, but her happiness after winning an Olympic gold medal is, itself, not evidence that Ms. Wieber is a “happy person.” The happiness of “happy people” does not depend on life circumstances. Rather, happy people seem to have personalities that allow them to find happiness even in the midst of adversity and challenging life conditions.

Myers and Diener (1995) identified four personality traits that characterize happy people: extraversion, locus of control, self-esteem and optimism. In the present research, we consider the possibility that people endowed with these personality traits habitually use humor as an adaptive strategy to maintain happiness. We present evidence that the relationship between each of the four “happy personality traits” and happiness is mediated by a self-enhancing humor style.

## Happiness and its Personality Correlates

Happiness is a complex construct. It is obvious and unmistakable; people generally know and can readily report whether they are happy or not (Lyubomirsky & Lepper, 1999) yet they find it difficult to precisely define (Freedman, 1978; Lyubomirsky, Tkach, & DiMatteo, 2006). Reflecting the intricacy of happiness, researchers generally define it as a multi-faceted construct, containing both a cognitive component—a *subjective* appraisal of life satisfaction, and an affective component—the relative preponderance of positive and negative emotions one experiences (e.g., Emmons & Diener, 1985; Lyubomirsky et al., 2006; Myers & Diener, 1995). Happy people, those high in subjective well-being (SWB), report more positive than negative thoughts and feelings about their lives (Myers & Diener, 1995).

Research on the correlates of happiness has long been guided by a “bottom-up” approach emphasizing the impact of external life events and demographic variables (Diener, 1984). Consistent with this bottom-up approach, research has shown that desirable life events (e.g., marriage, birth of a child, winning an Olympic gold medal) are associated with positive affect; whereas undesirable life events (e.g., divorce, health problems) are associated with negative affect (Stallings, Dunham, Gatz, Baker, & Bengtson, 1997). However, it appears that people adapt to positive and negative events, and over time approach previous levels of overall happiness (Brickman & Campbell, 1971; Headey & Wearing, 1992). In their classic study, for instance, Brickman, Coates, and Janoff-Bulman (1978) found that people adapted their level of happiness after even an extremely positive life event—winning the lottery—or an extremely negative life event —becoming paralyzed (see also Silver, 1982; Suh, Diener, & Fujita, 1996).

Similarly, demographic variables and quality of life indices (e.g., sex, race, age, education level, marital status, religious faith, income) appear to have only a modest relation with long-term reports of life satisfaction (e.g., Andrews & Withey, 1976; Argyle, 1999; Campbell, Converse, & Rodgers, 1976; Diener, Lucas, & Scollon, 2006). Campbell et al. (1976), for instance, reported that only 17% of the variance in life satisfaction could be explained by 10 demographic variables.

Collectively, these “bottom up” findings highlight the importance of an alternative “top-down” approach (Diener, 1984) that seeks to explain differences in happiness among people through stable personality traits. A large body of research has accumulated showing that personality traits are perhaps the most important correlates of happiness, explaining more of the total variance in happiness than demographic variables and quality of life indices (e.g., Andrews & Withey, 1976; DeNeve & Cooper, 1998; Diener, Suh, Lucas, & Smith, 1999; Gutiérrez, Jiménez, Hernández, & Puente, 2005).

Myers and Diener (1995) proposed that four personality traits, in particular, are strongly related to happiness: extraversion, locus of control, self-esteem, and optimism. These personality traits are thought to relate to happiness through two different mechanisms (Lyubomirsky, Tkach, & DiMatteo, 2006; McCrae & Costa, 1991). First, they can function through a temperamental mechanism; they predispose people to feel positive or negative emotions that influence self-reported happiness. Second, they can function through an instrumental mechanism; personality traits predispose people to seek out certain situations or activities, which in turn influence happiness.

### Extraversion

Among the four personality traits Myers and Diener (1995) identified, extraversion has received the most attention because it is one of the dimensions of the Five Factor Model of Personality, the predominant psychological paradigm for studying personality (e.g., Gosling & John, 1999; McCrae & Costa, 1999). In studies that group personality traits in terms of the Five Factor Model, extraversion has emerged as a particularly important and consistent correlate of happiness (e.g., Costa & McCrae, 1980; Diener & Lucas, 1999; Diener, Sandvik, Pavot, & Fujita, 1992; Gutiérrez et al., 2005; Hayes & Joseph, 2003). Hayes and Joseph (2003), for instance, reported that extraversion and neuroticism were the best predictors of scores on the Oxford Happiness Inventory.

Temperamentally, extraverts may be predisposed to experience positive emotions (Costa & McCrae, 1980) and experience positive events more intensely than those low in extraversion (Larsen & Ketelaar, 1989). Instrumentally, extraverts are happier because they have better social skills (Argyle & Lu, 1990a, 1990b) and experience greater social support (Lu, Shih, Lin, & Ju, 1997; Lyubomirsky et al., 2006).

### Locus of Control

Rotter (1966) described locus of control in terms of people’s expectations about the degree to which reinforcement that follows behavior is under internal control (i.e., contingent upon behavior, determined by one’s own actions) versus external control (i.e., not contingent upon behavior, determined by outside forces). People with an internal locus of control believe that their behavior causes outcomes that follow; thus they believe they have control over outcomes in most situations. In contrast, people with an external locus of control do not believe in the causal effect of their behavior on outcomes they experience in most situations; they attribute outcomes in most situations to external sources (e.g., luck, chance, fate or other people).

As Myers and Diener (1995) suggested, happy people are more likely to have an internal locus of control (Cummins & Nistico, 2002; Larson, 1989; Lu et al., 1997; Pannells & Claxton, 2008). Larson (1989) found that people who reported a higher internal locus of control in their daily lives also reported greater happiness. Consistent with this finding, Cummins and Nistico (2002) reviewed nine studies that examined the relationship between internal locus of control and life satisfaction. They reported correlations that ranged from .16 to .57 with an average correlation of .41. They also reported that the relationship between locus of control and happiness increases with age.

Lu et al. (1997) suggested that locus of control has primarily a direct, temperamental relationship with happiness, not mediated by social support like extraversion is. However, it is possible that locus of control has an instrumental link to happiness too. People high in internal locus of control tend to be more creative (Glover & Sautter, 1976; Pannells & Claxton, 2008). Thus, they may be better able to engage in cognitive strategies to attain happiness. Finally, Argyle (2001) suggested that people attend less to negative life events insofar as they have an internal locus of control.

### Self-Esteem

Self-esteem refers to the evaluative aspect of self-knowledge that is concerned with the degree to which people like themselves (Brown & Marshall, 2006). In other words, self-esteem captures the amount of personal value that individuals place on their self, as whole, or in different domains (Brown & Marshall, 2006). Self-esteem has been shown to have a strong relation to happiness (Cummins & Nistico, 2002; Diener, 1984) particularly in individualistic cultures (Diener & Diener, 1995). In general, people high in self-esteem are happier than those low in self-esteem (Argyle, 2001; Campbell, 1981; Cheng & Furnham, 2003). In addition, Lyubomirsky et al. (2006) found that self-esteem was positively correlated with optimism, and negatively correlated with hopelessness raising the possibility that self-esteem relates to happiness instrumentally, by predisposing people to view themselves as efficacious and to hold positive expectations about their lives (Baumeister, Campbell, Krueger, & Vohs, 2003).

### Optimism

Optimism is a dispositional quality that predisposes people to expect positive outcomes in life (Scheier & Carver, 1985). Research has shown that optimism is strongly related to overall happiness (e.g., Cummins & Nistico, 2002; Dember & Brooks, 1989) and life satisfaction (Lucas, Diener, & Suh, 1996) as well as greater positive affect (Seligman & Csikszentmihalyi, 2000) and less negative affect (Scheier & Carver, 1992). Optimism appears to relate to happiness instrumentally. When optimists encounter challenges they seem to employ more effective coping strategies, which in turn increases happiness (e.g., Taylor, Kemeny, Aspinwall, Schneider, & Rodriguez, 1992). Aspinwall and Taylor (1992), for instance, found that college freshmen higher in optimism engaged in more active coping strategies; those low in optimism employed more avoidant strategies.

## Humor Styles

Historically, researchers have regarded sense of humor has an exclusively adaptive and positive unitary disposition (Cann, Stilwell, & Taku, 2010). Martin, Puhlik-Doris, Larsen, Gray, and Weir (2003), however, distinguished between four humor styles or ways that people habitually use humor in daily life. Two of the humor styles, *affiliative* and *self-enhancing* are positive or beneficial to the self or others; the other two, *aggressive* and *self-defeating* are negative or detrimental to the self or others.

Martin et al.’s (2003) model has greatly elucidated the complex and often counter-intuitive relationship between humor and well-being. It has revealed that, depending on how it is used in daily life, humor can positively or negatively relate to a wide variety of manifestations of psychological well-being. See Cann and Collette (2014) and Martin (2007) for reviews.

People who have an affiliative humor style use humor to attain *interpersonal* or social rewards. They use humor to entertain others in order to enrich the quality of social relationships. People who have a self-enhancing humor style use humor to achieve *intrapersonal* rewards, that is, to enhance or maintain positive psychological well-being and distance themselves from adversity. They maintain a humorous outlook on life, coping with difficult circumstances by viewing them from a humorous perspective. Thus, self-enhancing humor is closely related to coping sense of humor (Cann et al., 2010; Martin et al., 2003).

Those with an aggressive humor style use humor, not to make interpersonal relationships more rewarding for the self and others, but rather as a means of criticizing or manipulating others. They tease and ridicule others to demonstrate their superiority over others, without concern for others’ well-being (Martin et al., 2003). Not surprisingly, the aggressive humor style has been shown to be detrimental to interpersonal relationships (e.g., Cann, Zapata, & Davis, 2011; Kuiper, Kirsh, & Leite, 2010). Finally, people who have a self-defeating humor style poke fun at their own weaknesses in order to ingratiate themselves to others. They also use humor as a means to avoid confronting problems and dealing with negative feelings (Stieger, Formann, & Burger, 2011).

## Humor Styles and Happiness

There has been an increasing interest in recent years in the relationship between humor styles and psychological well-being (Cann & Collette, 2014). Research has consistently shown that happiness is positively related to the two adaptive humor styles and negatively related to the two maladaptive humor styles (e.g., Ford, McCreight, & Richardson, 2014; Martin et al., 2003). Martin et al. (2003), for instance, reported that the Ryff (1989) measure of psychological well-being related positively to affiliative and self-enhancing humor styles and negatively to a self-defeating humor style. Furthermore, Martin et al. noted that anxiety and depression correlated negatively with affiliative and self-enhancing humor styles but positively with self-defeating humor style.

Cann et al. (2010) further demonstrated that the two self-directed humor styles (self-enhancing and self-defeating) are particularly strongly related to happiness. When they regressed measures of subjective well-being onto all four humor styles simultaneously only self-enhancing and self-defeating humor styles significantly predicted well-being. Similarly, Cann and Collette (2014) reported that among the four humor styles, only self-enhancing humor reliably contributed to stable positive affect ratings over a seven-day period.

In addition, researchers have found that positive and negative humor styles relate differently to personality traits associated with happiness. Yue, Hao, and Goldman (2010), for instance, found that affiliative and self-enhancing humor styles correlated positively with optimism, whereas aggressive and self-defeating humor styles correlated negatively with optimism. Similarly, self-enhancing humor and affiliative humor relate positively to self-esteem and judgments of self-competence. Aggressive and self-defeating humor styles, in contrast, are associated with lower self-esteem, and negative judgments of self-competence (Kuiper, Grimshaw, Leite, & Kirsh, 2004; Lui, 2012; Zeigler-Hill & Besser, 2011).

Finally, the two adaptive humor styles appear to mediate the relationship between personality traits and happiness. Lui (2012) found that the relationship between self-esteem and happiness was mediated by self-enhancing and affiliative humor styles but not by self-defeating or aggressive humor styles. Similarly, in a study of affective styles and happiness, Ford et al. (2014) found that “approach-oriented” people, those scoring high on Carver and White’s (1994) Behavioral Activating System (BAS) scale, were happier in part, because they habitually used a self-enhancing humor style in daily life. Thus, it appears that engaging in positive humor styles, perhaps especially a self-enhancing humor style, might be an instrumental strategy by which people attain happiness.

## The Present Research

The findings of previous research converge on a consistent and coherent story. Happy people tend to have four personality traits identified by Myers and Diener (1995): extraversion, high self-esteem, optimism, and internal locus of control that function temperamentally and instrumentally in the attainment of happiness. Also, happy people habitually engage in adaptive humor styles, particularly self-enhancing humor.

In the present research, we extend this literature by testing a new hypothesis about a mechanism by which these personality traits relate to happiness. Based on the premise that personality traits relate to happiness instrumentally, through an active pursuit of positive experiences, we hypothesized that the relationship between the four “happy personality traits” and happiness is mediated by positive humor styles. That is, people with the four happy personality traits are happier *because* they engage in self-enhancing humor and affiliative humor in daily life—they maintain a humorous outlook on life, viewing adversity from a humorous perspective, and they use humor in interpersonal settings to enrich the quality of social relationships.

## Method

### Participants and Procedure

We distributed a link to our study to 107 male and 87 female residents of the United States through Mechanical Turk (MTurk), an on-line participant pool sponsored by Amazon.com (for more information on data collection through MTurk, see Buhrmester, Kwang, & Gosling, 2011). Participants were paid $0.40 to complete the study. Participants ranged in age from 20 to 67 years, with a mean of 32.59 (*SD* = 9.56). The mean age was 30.76 (*SD* = 8.15) for males and 34.95 (*SD* = 10.71) for females. There were 7 African-Americans, 107 Asians, 8 Hispanics, 2 Native Americans, 66 Whites, and 4 people who indicated their race as “other.”

Upon clicking a link to the study, participants accessed the study in Qualtrics. Participants first encountered the following instructions: “You are invited to participate in a study designed to determine how different personality traits are related to one another. For this study, you will complete six different personality surveys and five demographic questions.” After providing informed consent, participants completed the following measures of the “happy personality traits”: the Extraversion scale from Eysenck and Eysenck’s (1975) Personality Questionnaire (EPQ-E), Rotter’s (1966) Locus of Control scale (LOC), Rosenberg’s (1965) Self-esteem Scale (RSES), and Scheier, Carver, and Bridges’ (1994) Life Orientation Test-Revised (LOT-R). Participants then completed Martin et al.’s (2003) Humor Styles Questionnaire (HSQ), and Hills and Argyle’s (2002) Oxford Happiness Questionnaire (OHQ).

### Measures

#### Eysenck’s Personality Inventory-Extraversion (EPI-E)

Participants responded “yes” (coded as “1”) or “no” (coded as “0”) to 24 questions from Eysenck & Eysenck’s (1964) EPI-E designed to assess the degree to which they were generally extraverted or introverted (e.g., “Generally, do you prefer reading to meeting people?”). Higher scores indicate greater extraversion. Cronbach’s alpha for the scale was .68.

#### Locus of Control Scale (LOC)

Rotter’s (1966) LOC consisted of 29 pairs of statements. One statement of each pair reflected an internal locus of control (e.g., “People’s misfortunes result from the mistakes they make.”), and one reflected an external locus of control (e.g., “Many of the unhappy things in people’s lives are partly due to bad luck.”). Participants indicated which statement of each pair they agreed with more. We coded internal LOC statements as “1” and external LOC statements as “0”, thus, higher scores indicated greater internal LOC. Cronbach’s alpha was .65.

#### Self-Esteem Scale (RSES)

Rosenberg’s (1965) self-esteem scale consists of 10 items (e.g., “I feel that I'm a person of worth, at least on an equal plane with others.”). Participants responded to each item on a scale ranging from 1 (strongly disagree) to 4 (strongly agree). We averaged responses to the 10 items to form an aggregate measure of self-esteem. Higher scores indicated higher self-esteem. Cronbach’s alpha for the 10-item scale was .80.

#### Life Orientation Test-Revised (LOT-R)

Scheier, Carver, and Bridges’ (1994) revised Life Orientation Test consists of 10 statements; 6 were designed to measure optimism (e.g., “In uncertain times, I usually expect the best.”). Participants indicated their agreement with each statement on a 5-point scale ranging from 1 (strongly disagree) to 5 (strongly agree). We averaged responses to the six items to form an aggregate measure of optimism. Higher scores indicated greater dispositional optimism. Cronbach’s alpha for the 6-item scale was .66.

#### Humor Styles Questionnaire (HSQ)

Martin et al.’s (2003) HSQ consists of four 8-item subscales that measure the degree to which people habitually engage in affiliative humor (e.g., ‘‘I enjoy making people laugh”), self- enhancing humor (e.g., ‘‘Even when I’m by myself, I’m often amused by the absurdities of life’’), aggressive humor (e.g., ‘‘If I don’t like someone, I often use humor or teasing to put them down’’), and self-defeating humor (e.g., ‘‘I will often get carried away in putting myself down if it makes my family or friends laugh’’). Participants indicated their agreement with each item using a 7-point scale ranging from 1 (strongly disagree) to 7 (strongly agree). We averaged the responses for the four items of each subscale for each participant. Cronbach’s alpha was .77 for affiliative humor, .73 for self-enhancing humor, .58 for aggressive humor, and .83 for self-defeating humor.

#### Oxford Happiness Questionnaire (OHQ)

Hills and Argyle’s (2002) Oxford Happiness Questionnaire (OHQ) consists of 29 items designed to measure enduring happiness or subjective well-being (e.g., “I am well satisfied with everything in my life.”). Participants indicated their agreement with each statement using a 6-point scale ranging from 1 (strongly disagree) to 6 (strongly agree). We averaged responses to the 29 items to form an aggregate measure of dispositional happiness. Higher scores indicated higher happiness. Cronbach’s alpha was .92.

## Results

### Descriptive Statistics

Table 1 displays the correlations among the four happy personality traits, the four humor styles and happiness.

**Table 1:** Correlations and Descriptive Statistics for Measures of the Four Happy Personality Traits, Measures of the Four Humor Styles and Scores on the OHQ

Scale	1	2	3	4	5	6	7	8	9	*M*	*SD*
1. EPI-E	----									11.34	3.94
2. LOC	.23**	----								12.90	3.84
3. RSES	.13	.29**	----							2.95	0.46
4. LOT-R	.20**	.33**	.61**	----						3.29	0.67
5. SE-H	.32**	.18*	.30**	.20**	----					4.77	0.85
6. AFF-H	.14*	.17*	.29*	.27**	.21**	----				4.56	1.03
7. SD-H	.10	-.16*	-.38**	-.36**	.07	-.30**	----			4.19	1.17
8. AGG-H	.26**	-.10	-.19**	-.26**	-.07	-.08	.32**	----		3.73	0.84
9. OHQ	.37**	.48**	.71**	.62**	.35**	.41**	-.21**	-.14*	----	4.05	0.73

*Note.* EPI-E = Extraversion, LOC = Locus of Control, RSES = Self-esteem, LOT-R = Optimism, SE-H = Self-enhancing Humor Style, AFF-H = Affiliative Humor Style, SD-H = Self-defeating Humor Style, AGG-H = Aggressive Humor Style, OHQ = Happiness.

*p < .05. **p < .01.

Consistent with previous research, happiness correlated positively with each of the four happy personality traits, and with self-enhancing and affiliative humor styles. Finally, happiness correlated negatively with self-defeating and aggressive humor styles. Also, in keeping with our hypothesis, self-enhancing humor and affiliative correlated positively with each of the four happy personality traits. Thus, people higher in extraversion, internal locus of control, self-esteem and optimism also reported that they habitually use self-enhancing humor and affiliative humor in daily life.

Also, like Cann et al. (2010), we regressed happiness simultaneously on all four humor styles. Unlike Cann et al., however, both self-enhancing humor and affiliative humor emerged as significant predictors of happiness (β = .29, *SE* = 0.06, *t* = 4.51, *p* < .001; β = .31, *SE* = 0.05, *t* = 4.62, *p* < .001 respectively). Thus, both self-enhancing humor and affiliative humor significantly related to happiness when controlling for each of the other humor styles. In contrast, self-defeating and aggressive humor styles did not significantly predict happiness when controlling for the effects of the other humor styles (β = -.12, *SE* = 0.04, *t* = 1.65, *p* = .10; β = -.06, *SE* = 0.06, *t* = -0.93, *p* = .35 respectively).

### Mediation Analyses

**Figure 1:**
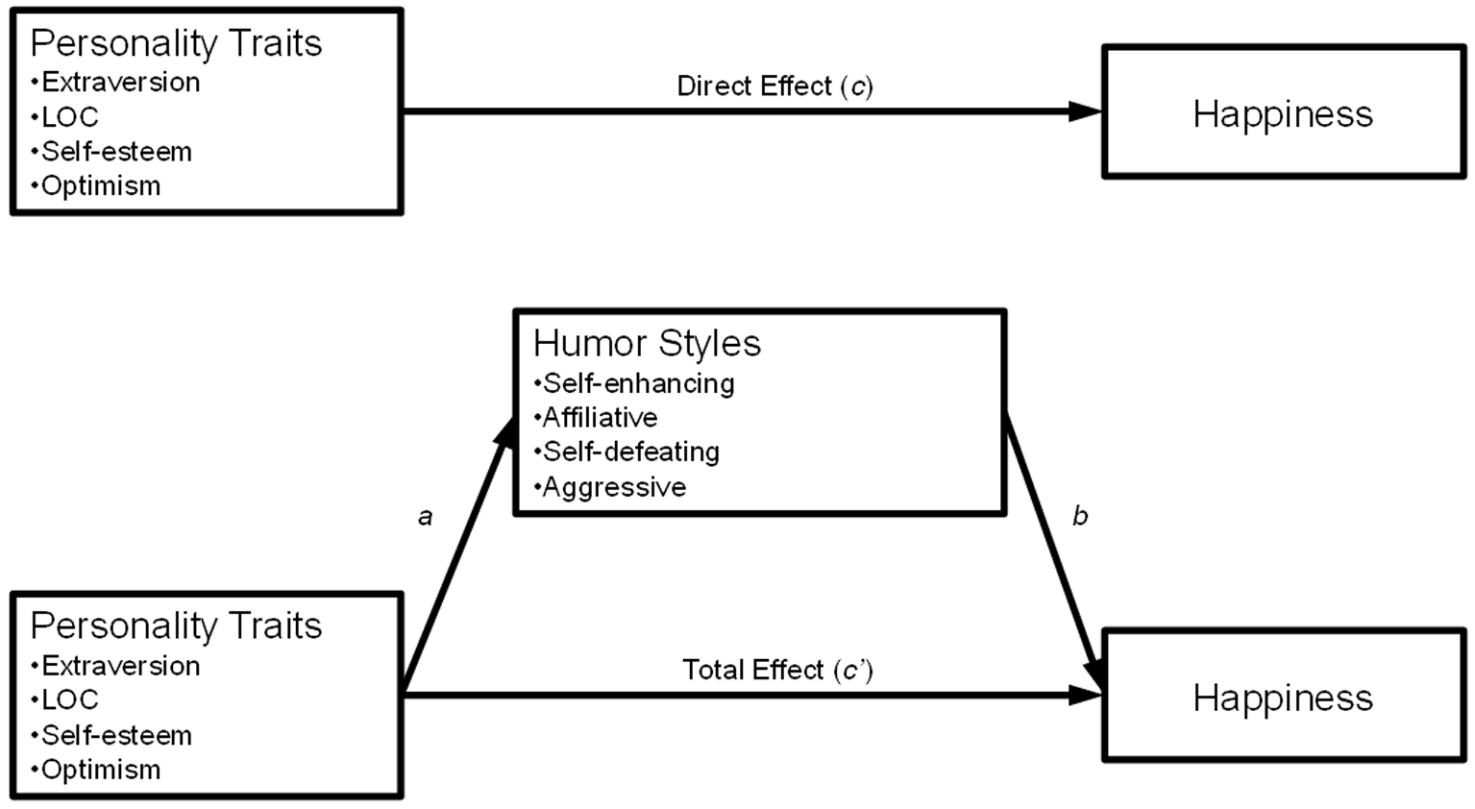
Path Model illustrating our mediation hypotheses including labels for direct and indirect paths. We hypothesized that the relationship between personality traits and happiness is mediated by self-enhancing humor.

We conducted a series of four mediation analyses to test our hypothesis that the relationship between the four happy personality traits and happiness is mediated by the four humor styles, with a particular interest in self-enhancing humor and affiliative humor. More specifically, we tested a path model wherein each of the happy personality traits acted as predictor variables, humor style acted as a single mediator, and happiness as the outcome variable. That is, a separate analysis was conducted for each humor style. We evaluated both direct paths between the personality traits and happiness and the indirect paths by which personality traits relate to happiness through humor style. We present the path model in Figure 1.

We used the PROCESS macro for SPSS to conduct the mediation analysis. This macro uses a bootstrapping procedure to obtain confidence intervals for the indirect effect (Hayes, 2013), and has been demonstrated to have greater power than the causal steps procedures developed by Baron and Kenny, (e.g., Baron & Kenny, 1986). The bootstrapping procedure tests whether the indirect effect (i.e., the path from the personality traits to happiness through a humor style) is different from zero by providing a 95% confidence interval for the population value of the indirect effect (Preacher & Hayes, 2004). If zero is not in the 95% confidence interval the indirect effect is significant at *p* < .05.

Finally, we conducted separate mediation analyses for each of the four personality traits. However, a seeding procedure implemented in the PROCESS macro linked the analyses, ensuring that each bootstrapping process begins with the same random number, in turn causing the confidence intervals between the four models to have the same bounds (see Hayes, 2013, for an extended discussion). Thus, although these analyses were conducted separately in PROCESS, they can be considered as parts of the same larger mediation analysis. In sum, our analysis consists of four models that examine the mediating role of self-enhancing humor, affiliative humor, self-defeating humor and aggressive humor respectively. We present summary statistics for our mediation models in Table 2, and further describe analyses for Models 1 and 2^i^.

**Table 2:** Summary of Mediation Analysis for Personality Traits and Happiness (N = 195; 10,000 bootstraps)

Predictor Variable (PV)	Mediating Variable (M)	Criterion Variable (CV)	Model Number	Effect of PV on M (a)	Effect of M on CV (b)	Direct effect (c’)	Indirect effect (bias corrected intervals) (a)(b): 95% CI	Total effect (c)
**Extraversion**	SE-H	Happiness	1	.07***	.23***	0.05***	.02: [.0051, .0325]	0.07***
	AFF-H		2	.04*	.26***	0.06	.01: [-.0004, .0238]	0.07***
	SD-H		3	.03	-.15***	0.07	-.00: [-.0139, .0015]	0.07***
	AGG-H		4	.06***	-.22***	0.08***	-.01: [-.0238, -.0053]	0.07***
**LOC**	SE-H	Happiness	1	.04*	.24***	0.08***	.01: [.0006, .0222]	0.09***
	AFF-H		2	.05*	.24***	0.08***	.01: [.0012, .0245]	0.09***
	SD-H		3	-.05*	-.08*	0.09***	.00: [.0002, .0132]	0.09***
	AGG-H		4	-.02*	-.09*	0.09***	.00: [-.0008, .0111]	0.09***
**Self-esteem**	SE-H	Happiness	1	.55***	.13**	1.05***	.07: [.0197, .1564]	1.12***
	AFF-H		2	.66***	.16**	1.02***	.10: [.0355, .1998]	1.12***
	SD-H		3	-.97***	.05**	1.17***	-.05: [-.1260, .0165]	1.12***
	AGG-H		4	-.34**	-.01	1.12***	.00: [-.0252, .0566]	1.12***
**Optimism**	SE-H	Happiness	1	.26***	.20***	0.62***	.05: [.0090, .1183]	0.67***
	AFF-H		2	.41***	.19***	0.59***	.08: [.0241, .1559]	0.67***
	SD-H		3	-.62***	.01	0.68***	-.01: [-.0504, .0382]	0.67***
	AGG-H		4	-.33***	.01	0.68***	-.00: [-.0457, .0364]	0.67***

*p < .05. **p < .01. ***p < .001.

As shown in Table 2, a very similar pattern of results emerged across the four happy personality traits as predictors of happiness with self-enhancing humor (Model 1) and affiliative humor (Model 2) serving as mediators. Therefore, we describe the findings from both models across each of the four happy personality traits.

#### Extraversion

The total amount of variance in happiness explained by extraversion and self-enhancing humor in Model 1 was significant, *R*^2^ = .38, *p* < .05. In addition, evidence of mediation emerged; extraversion had a significant positive indirect link with happiness through self-enhancing humor, as the 95% confidence interval did not contain zero. The total amount of variance in happiness explained by extraversion and affiliative humor in Model 2 was significant (but less than Model 1), *R*^2^ = .14, *p* < .05. However, unlike Model 1, affiliative humor did not appear to mediate the relationship between extraversion and happiness. The 95% confidence interval did include zero.

#### Locus of Control

The total amount of variance in happiness explained by locus of control and self-enhancing humor in Model 1 was significant, *R*^2^ = .23, *p* < .05. In addition, evidence of mediation emerged. Locus of control had a significant positive indirect link with happiness through self-enhancing humor; the 95% confidence interval did not contain zero. The total amount of variance in happiness explained by locus of control and affiliative humor in Model 2 was the same as in Model 1, *R*^2^ = .23, *p* < .05. And, like Model 1, affiliative humor emerged as a significant mediator; the 95% confidence interval did not include zero.

#### Self-Esteem

Similar results emerged for the two models in which self-esteem predicted happiness. The total amount of variance in happiness explained by self-esteem and self-enhancing humor in Model 1 was significant, *R*^2^ = .51, *p* < .05. And, self-enhancing humor significantly mediated the relationship between self-esteem and happiness; the 95% confidence interval did not contain zero. The findings for Model 2, which included affiliative humor as a mediator were identical to those of Model 1. The total amount of variability in happiness explained by the model was *R*^2^ = .51, and affiliative humor emerged as a significant mediator; the 95% confidence intervals did not contain zero.

#### Optimism

Finally, the total amount of variance in happiness explained by optimism and self-enhancing humor in Model 1 was significant, *R*^2^ = .39, *p* < .05. And, optimism had a significant indirect link with happiness through self-enhancing humor; the 95% confidence interval did not contain zero. Similarly, optimism and affiliative humor in Model 2 explained a significant amount of the variance in happiness, *R*^2^ = .38, *p* < .05. And, like Model 1, affiliative humor emerged as a significant mediator; the 95% confidence interval did not include zero.

## Discussion

The results of our study contribute to the findings from several different lines of research. First, bivariate correlations replicate previous findings on the relationship between the happy personality traits identified by Myers and Diener (1995) and happiness. People reported being happy insofar as they were high in extraversion, personal (internal) control, self-esteem and optimism. Second, bivariate correlations replicated research showing that happiness is positively correlated with self-enhancing and affiliative humor styles (e.g., Martin et al., 2003).

Unlike previous research (e.g., Cann & Etzel, 2008), however, we did not find that self-enhancing humor more strongly related to happiness than affiliative humor when examined in a regression equation that simultaneously controlled for the effect of each of the other humor styles. We found that both self-enhancing humor and affiliative humor predicted happiness when controlling for the other humor styles. This discrepancy could be due to differences between studies in the magnitude of the correlation between self-enhancing and affiliative humor styles. In our study, the correlation was relatively modest (r = .21), whereas Cann and Etzel found a much larger correlation (r = .43). Thus, in our study, there appears to have been less conceptual overlap between the two adaptive humor styles. Consequently, when we simultaneously controlled for the effect of each of the other humor styles in a regression analysis, we were better able to detect a unique association between affiliative humor style and happiness.

In addition, the mediation analyses support our hypothesis and contribute to a growing body of research showing that humor styles mediate the relationship between personality and happiness (e.g., Ford et al., 2014; Lui, 2012). Positive humor styles mediated the relationship between each of the four happy personality traits identified by Myers and Diener (1995) and happiness. Consistent with the theory that personality traits relate to happiness instrumentally, our findings suggest that people who are high in extraversion, internal locus of control, optimism, and self-esteem have developed adaptive strategies of using humor in daily life, which in turn help make them happy. They experience greater happiness because they are better at finding strategies to regulate their emotions (Baumeister et al., 2003), and the habitual use of positive humor is one of those strategies. Happy people may be adept at using positive humor styles as a means by which they frame or appraise life events to form positive, self-affirming views of the self (Kuiper & McHale, 2009). Indeed, people protect their psychological well-being by using self-enhancing humor as a means of reframing stressors in a more positive, light-hearted way (e.g., Cann & Etzel, 2008; Cann et al., 2010; Martin et al., 2003)

The one surprising exception to this pattern is that affiliative humor did not significantly mediate the relationship between extraversion and happiness. Perhaps the temperamental link between extraversion and happiness is particularly strong (e.g., Argyle & Lu, 1990a; Brebner, Donaldson, Kirby, & Ward, 1995; Costa & McCrae, 1980; Pavot, Diener, & Fujita, 1990), and thus overwhelms or obscures the instrumental influence of mediator variables, especially those that do not have a strong influence.

Collectively, our findings contribute to the broader literature on the relationship between humor styles, personality and well-being (e.g., Besser, Luyten, & Mayes, 2012; Cann, Norman, Welbourne, & Calhoun, 2008; Dozois, Martin, & Bieling, 2009; Kuiper & McHale, 2009; Zeigler-Hill & Besser, 2011). One’s personality appears to function as a lens that colors the way people view themselves and social settings. The use of positive or negative forms of humor seems to follow from the valence of that general lens and thus contributes to a positive or negative sense of well-being.

Kuiper and McHale (2009), for instance, found that humor styles mediate the relationship between beliefs about the self and low self-esteem. People who disproportionately focus on their negative attributes are particularly prone to engage in self-defeating humor and thus experience lower self-esteem. In contrast, people who have more positive self-beliefs engage in more affiliative humor, which in turn increases self-esteem and decreases depression. These results make sense in light of the Sociometer Theory of self-esteem (Leary & Baumeister, 2000), which suggests that self-esteem functions as a gauge of the relational value others hold for you. That is, if others hold you in high regard, and are motivated to include you in their activities, you have high relational value. When individuals perceive that they have high relational value, their feelings of self-worth are bolstered, which results in high self-esteem. Our results also support the sociometer theory. More specifically, self-esteem was positively associated with self-enhancing and affiliative humor, whereas self-esteem was negatively associated with self-defeating and aggressive humor styles. Therefore, positive humor styles may be seen as desirable traits by others, and may function to foster relationships, whereas the negative humor styles may be seen as undesirable, or even offensive.

Additionally, the present findings have potentially interesting implications for future research on the relationship between humor styles and happiness. Like other studies investigating the correlates of humor styles, the present research treated humor styles as a personality variable in a non-experimental, correlational study. The findings of the present research, however, raise the possibility that inducing people to engage in adaptive forms of humor can have positive psychological outcomes. Future research could expand on the present findings by treating humor styles as an independent variable in an experimental design (see Maiolino & Kuiper, 2016; Samson & Gross, 2012) to investigate the effects of engaging in adaptive and maladaptive forms of humor on momentary (rather than trait) expressions of happiness. On the basis of the present findings, we hypothesize that engaging in adaptive forms of humor would enhance momentary happiness (i.e., increase positive affect, decrease negative affect), whereas engaging in maladaptive forms of humor would have the opposite effects.

### Limitations

Our study makes a new contribution to our understanding of the relationship between personality traits, humor styles and happiness. It does, however, have limitations. For instance, Kashdan (2004) criticized our measure of happiness, the Oxford Happiness Questionnaire, for being too closely related to self-esteem and extraversion among other personality constructs. This conceptual overlap could inflate correlations between the OHQ and our measures of self-esteem and extraversion, or potentially lead to suppressor effects (Friedman & Wall, 2005).

Although we agree that the conceptual overlap between the OHQ and personality variables of interest is a problematic, we do not believe that it has compromised the test of our hypothesis. First, Steel, Schmidt, and Shultz (2008) argued that the EPI overlaps less conceptually with positive affect in comparison to Eysenck and Eysenck’s (1975) EPQ. Accordingly, the EPI should be less vulnerable to inflated correlations with our measure of happiness. Indeed, the correlation between the EPI and the OHQ in our study was .37. Second, even if the bivariate correlations were inflated, there is no reason to believe that the mediation models testing our hypotheses would be compromised by the problems Kashdan (2004) has raised about the OHQ.

Additionally, it should be noted that this study is correlational in nature, and relies on the use of self-report. Therefore, the data are subject to sources of bias such as social desirability. Furthermore, the internal consistency of some of the scales used was relatively low (e.g., *α =* .66). Although the low alpha values warrant caution, they could be due, in part, to heterogeneity of the underlying constructs, and the number of items in the measures (Nunnally & Bernstein, 1994; Tavakol & Dennick, 2011).

Finally, it should be noted that although we tested an underlying process model wherein humor styles mediate the relationship between personality and happiness, other possibilities exist. For example, Cann and Etzel (2008) found that engaging in a self-enhancing humor style increases optimism, hope and happiness, which in turn decreases perceived stress in one’s life. Self-enhancing humor seems to foster the development or activation of positive personality qualities that are associated with coping with the stresses of life. As Cann et al. (2010) suggested, it is possible that there are complex connections between stable personality traits, dispositional happiness and psychological responses to life events, and that humor styles play multiple roles in those connections. For instance, humor styles might play a mediating role in the relationship between personality traits and dispositional happiness (e.g., Ford et al., 2014). However, the relationship between humor styles and psychological responses to life events (e.g., stressors) can be mediated by other (instrumental) psychological variables or behaviors.

### Conclusion

Personality is an important determinant of one’s happiness. As Myers and Diener (1995) stated, happy people tend to be extraverted, and optimistic; they tend to have a sense of locus of control, and high self-esteem. People with these personality traits engage in strategies that are instrumental in producing happiness. The present study reveals that the use of positive forms of humor, self-enhancing humor and affiliative humor, represents one such strategy.
